# Preliminary Investigation of the Safety of Escalating Cannabinoid Doses in Healthy Dogs

**DOI:** 10.3389/fvets.2020.00051

**Published:** 2020-02-11

**Authors:** Dana Vaughn, Justyna Kulpa, Lina Paulionis

**Affiliations:** Canopy Animal Health, Canopy Growth Corporation, Toronto, ON, Canada

**Keywords:** cannabinoids, CBD—cannabidiol, THC—tetrahydrocannabinol, safety, adverse events, canine

## Abstract

**Objective:** To determine the safety and tolerability of escalating doses of three cannabis oil formulations, containing predominantly CBD, THC, or CBD and THC (1.5:1) vs. placebo in dogs.

**Design:** Randomized, placebo-controlled, blinded, parallel study.

**Animals:** Twenty healthy Beagle dogs (10 males, 10 females).

**Methods:** Dogs were randomly assigned to one of five treatment groups (*n* = 4 dogs per group balanced by sex): CBD-predominant oil, THC-predominant oil, CBD/THC-predominant oil (1.5:1), sunflower oil placebo, medium-chain triglyceride oil placebo. Up to 10 escalating doses of the oils were planned for administration via oral gavage, with at least 3 days separating doses. Clinical observations, physical examinations, complete blood counts, clinical chemistry, and plasma cannabinoids were used to assess safety, tolerability, and the occurrence of adverse events (AEs). AEs were rated as mild, moderate, or severe/medically significant.

**Results:** Dose escalation of the CBD-predominant oil formulation was shown to be as safe as placebo and safer than dose escalation of oils containing THC (CBD/THC oil or THC oil). The placebo oils were delivered up to 10 escalating volumes, the CBD oil up to the tenth dose (640.5 mg; ~62 mg/kg), the THC oil up to the tenth dose (597.6 mg; ~49 mg/kg), and the CBD/THC oil up to the fifth dose (140.8/96.6 mg CBD/THC; ~12 mg/kg CBD + 8 mg/kg THC). AEs were reported in all dogs across the five groups and the majority (94.9%) were mild. Moderate AEs (4.4% of all AEs) and severe/medically significant AEs (0.8% of all AEs) manifested as constitutional (lethargy, hypothermia) or neurological (ataxia) symptoms and mainly occurred across the two groups receiving oils containing THC (CBD/THC oil or THC oil).

**Conclusions and clinical significance:** Overall, dogs tolerated dose escalation of the CBD oil well, experiencing only mild AEs. The favorable safety profile of 10 escalating doses of a CBD oil containing 18.3–640.5 mg CBD per dose (~2–62 mg/kg) provides comparative evidence that, at our investigated doses, a CBD-predominant oil formulation was safer and more tolerated in dogs than oil formulations containing higher concentrations of THC.

## Introduction

As a result of changing cannabis regulatory frameworks and social perceptions globally, there is a renewed interest in the potential therapeutic properties of cannabinoids across multiple stakeholders, including the veterinary community. Currently, there are no authorized veterinary drugs containing cannabinoids in the United States (U.S.) or Canada and state or federal laws legalizing the use of medical cannabis in either jurisdiction do not apply to uses in animals (1, 2). Notwithstanding these restrictions, over half (55%) of U.S.-based veterinarians who responded to a 2018 online survey had clients inquire weekly or monthly about the use of CBD products in animals (3). Online surveys also provide data that pet owners in the U.S. and Canada have purchased cannabis products for their pets most commonly for the management of pain, inflammation, and anxiety (4, 5). The evidence suggests there is a growing interest in the potential therapeutic uses of cannabinoids in companion animals.

While there are existing reviews on the safety and toxicology of cannabinoids, namely CBD (6–8) and THC (9), the data are primarily based on studies conducted in rodents and humans. Existing data suggest that there are differences in the metabolism of cannabinoids across species, with different CBD/THC metabolic profiles observed across rodents, dogs, monkeys, and humans (10–12). Not surprisingly, differences in the behavioral/physiological effects of cannabinoids across species have also been reported (11, 13).

While minimal, there are published data on the safety and efficacy of cannabinoids in companion animals. Dogs have been used in the course of developing the drug safety profile of Cesamet™ (nabilone, a synthetic THC; oral) and Sativex® (a combination of plant-based CBD and THC; buccal spray), both approved drugs in the U.S. and/or Canada for chemotherapy-induced nausea and vomiting (Cesamet™), or spasticity or pain in multiple sclerosis or advanced cancer (Sativex®) (14, 15). Published studies in dogs also exist on the pharmacokinetics of CBD (oral; 2–12 mg/kg) or THC (oral; 1.5 mg/kg) (16–19) and on the safety and/or efficacy of orally administered CBD (17, 20, 21) or cannabis extracts (11) for 4–56 weeks.

The primary objective of this study was to determine the safety of three cannabis oil formulations, predominant in CBD, THC, or CBD and THC (1.5:1) in dogs. Since a hallmark dosing strategy for cannabis initiation is to “start low and go slow” so as to avoid AEs associated with THC (22), a slow upward dose titration was used. A secondary objective was to determine blood levels of CBD, THC, and their metabolites, namely 7-carboxy-CBD (7-COOH-CBD) and 11-hydroxy-THC (11-OH-THC) at higher dose levels of CBD (>50 mg/kg) and THC (>30 mg/kg).

## Materials and Methods

### Study Design

The study was randomized, placebo-controlled, and blinded. Twenty-four healthy Beagle dogs were acclimated to study conditions for 24 days prior to treatment allocation. Twenty healthy adult purpose-bred Beagle dogs (age range = 3.0–7.8 years; weight range = 10–14 kg) were randomized to one of five treatment groups with four dogs per group (two males, two females): (i) CBD-predominant oil; (ii) THC-predominant oil; (iii) CBD/THC-predominant oil (1.5:1); (iv) medium-chain triglyceride (MCT) oil placebo; or (v) sunflower (SF) oil placebo.

Up to 10 escalating doses of the oils were planned for administration (Table 1), with at least 3 days separating doses. An absolute quantity of cannabinoids (mg) (Table 1) was administered to the dogs in each treatment group; as such, the resultant mg/kg cannabinoid dose slightly varied within each group due to body weight differences (Table 2).

**Table 1:** Dose volumes and CBD/THC quantities delivered to dogs across treatment groups.

**In medium-chain triglyceride oil**								**In sunflower oil**				
**Dose No**.	**Placebo—MCT oil (*****n*** **=** **4)**	**CBD oil (*****n*** **=** **4)**			**THC oil (*****n*** **=** **4)**			**Dose No**.	**Placebo—SF oil (*****n*** **=** **4)**	**CBD/THC oil (*****n*** **=** **4)**		
	**Vol (mL)**	**Vol (mL)**	**CBD (mg)**	**THC (mg)**	**Vol (mL)**	**CBD (mg)**	**THC (mg)**		**Vol (mL)**	**Vol (mL)**	**CBD (mg)**	**THC (mg)**
1	0.5^a^	1	18.3	0.7	1	ND^b^	24.9	1	2.5^a^	2.5	17.6	12.1
2	1^a^	2.5	45.8	1.7	2.5	*^b^	62.3	2	5^a^	5	35.2	24.2
3	2.5	5	91.5	3.5	3.3	*	82.2	3	10	10	70.4	48.3
4	5	7.5	137.3	5.2	4.4	*	109.6	4	15	15	105.6	72.5
5	10	10	183.0	6.9	5.8	*	144.4	5	20	20^c^	140.8	96.6
6	15	15	274.5	10.4	7.7	*	191.7	6	25	-	-	-
7	20	20	366.0	13.8	10.2	*	254.0	7	30	-	-	-
8	25	25	457.5	17.3	13.5^c^	*	336.2	8	35	-	-	-
9	30	30	549.0	20.7	18^c^	*	448.2	9	40	-	-	-
10	35	35	640.5	24.2	24^c^	*	597.6	10	45	-	-	-

(-), doses not administered; AE, adverse event; CBD, cannabidiol; MCT, medium-chain triglyceride; ND, not detected; SF, sunflower; THC, delta-9-tetrahydrocannabinol; Vol, volume.

a Placebo controls were on a dosing schedule such that two placebo doses were administered prior to the start of test formulation dosing. This was to ascertain tolerability to escalating volumes.

b CBD was “not detected” in the cannabinoid analysis (mg/mL) of the THC oil formulation; the symbol (*) indicates that CBD quantities at higher volumes of the formulation are unknown.

c *n = 3 dogs*.

**Table 2:** Corresponding range of mg/kg cannabinoid doses achieved in dogs across groups.

	**CBD oil**		**THC oil^**a**^**	**CBD/THC oil**	
**BSN weight range^**b**^**	**9.9–10.9 kg**		**11.4–13.3 kg**	**10.5–12.5 kg**	
**Dose No**.	**CBD (mg/kg)**	**THC (mg/kg)**	**THC (mg/kg)**	**CBD (mg/kg)**	**THC (mg/kg)**
1	1.7–1.8	0.06–0.07	1.9–2.2	1.4–1.7	0.97–1.2
2	4.2–4.6	0.16–0.17	4.7–5.5	2.8–3.4	1.9–2.3
3	8.4–9.2	0.32–0.35	6.2–7.2	5.6–6.7	3.9–4.6
4	12.6–13.9	0.48–0.53	8.2–9.6	8.4–10.1	5.8–6.9
5	16.8–18.5	0.63–0.70	10.9–12.7	11.3–13.4	7.7–9.2
6	25.2–27.7	0.95–1.1	14.4–16.8	-	-
7	33.6–37.0	1.3–1.4	19.1–22.3	-	-
8	42.0–46.2	1.6–1.7	25.3–29.5	-	-
9	50.4–55.5	1.9–2.1	33.7–39.3	-	-
10	58.8–64.7	2.2–2.4	44.9–52.4	-	-

(-), doses not administered; BSN, baseline.

a CBD was not detected in the THC oil formulation; as such, mg/kg CBD dosing not applicable.

b *Weight range of dogs and absolute quantity of cannabinoids per dose (Table 1) were used to calculate mg/kg dose*.

Two different placebo oils were used since the cannabinoid oils included either SF or MCT oil as solvents. Moreover, the amounts delivered across the two placebo oils differed to match the volumes administered with the cannabinoid oils. The dosing schedule of the placebo oils was such that two placebo doses were administered prior to the start of cannabinoid oil dosing to ascertain tolerability to increasing volumes.

Treatments were administered to fasted dogs by oral gavage. Each cannabinoid or placebo oil administration was followed by a 10 mL water flush to ensure full uptake. A small wet meatball was given immediately after dosing to disrupt negative association with repeated oral gavage. If an animal was observed vomiting within 30 min of test or placebo administration, the dose was re-administered.

#### Randomization and Treatment Allocation

Randomization was stratified by sex and conducted using a random number generator in Microsoft EXCEL® 2016 (Microsoft Corporation, Redmond, WA). Cannabinoid and placebo oil formulations were also randomly assigned a code name using a random number generator in Microsoft EXCEL® 2016. Once the dogs were assigned to a group (Groups 1 through 5), the study coordinator randomly allocated each group to a coded product by drawing lots. All technicians collecting data and administering the investigational products were blinded to treatment allocation. All bottles containing the cannabinoid or placebo oil formulations were over-labeled with opaque white labels. Information about treatment groups and their respective treatment conditions were securely kept in the VivoCore Inc. archive room for the duration of the study.

### Description of Interventions

The cannabinoid oils (CBD-, THC-, or CBD/THC-predominant oils) and placebo oils (SF or MCT oils) were acquired from Tweed Inc. (Smiths Falls, ON, Canada) and stored between 19.6 and 21.9°C. The cannabis plants used to prepare the cannabinoid oils were grown indoors under tightly controlled environmental conditions. Within one lot, all plants were genetically identical. Upon harvest, plant material was trimmed, dried, and extracted. Extraction was performed by super-critical carbon dioxide, and the extracted resin was diluted with a food-grade oil (SF or MCT oil) to the target concentration: 18.3 mg/mL CBD in the CBD-predominant oil, 24.9 mg/mL THC in the THC-predominant oil, and 7.0 mg/mL CBD + 4.8 mg/mL THC in the CBD/THC-predominant oil.

An independent laboratory (RPC, Fredericton, NB) analyzed the composition of the cannabinoid oil formulations using validated methods. Levels of phytocannabinoids and terpenes in the oil formulations are outlined in Table 3. Solvent extraction and high-performance liquid chromatography with diode-array detection (HPLC-DAD) were used for cannabinoid analyses (accuracy: 90–113%; precision: 5.6–12.8%). Solvent extraction and gas chromatography/mass selective detector (GC-MSD) were used for terpene analyses (accuracy: 74–106%; relative standard deviation: 3.2–9.4%).

**Table 3:** Select cannabinoid and terpene analysis of the cannabis oil formulations.

**Constituent**	**CBD oil (with MCT oil)**	**THC oil (with MCT oil)**	**CBD/THC oil (with SF oil)**
**Cannabinoids (mg/mL)**^**a**^			
CBD	18.3	ND	7.0
Delta-9-THC	0.7	24.9	4.8
CBDA	0.6	ND	<RL
Delta-9-THCA	ND	<RL	<RL
CBG	<RL	2.5	<RL
CBGA	ND	ND	<RL
CBN	ND	1.3	<RL
CBC	0.8	1.5	<RL
Terpenes (%)	21 terpenes below RL (0.01%)^b^	Caryophyllene (0.02%); remaining 8 terpenes below RL (0.01%)^c^	9 terpenes below RL (0.01%)^c^

CBC, cannabichromene; CBD, cannabidiol; CBDA, cannabidiolic acid; CBG, cannabigerol; CBGA, cannabigerolic acid; CBN, cannabinol; MCT, medium-chain triglyceride; ND, not detectable; RL, reporting limit; SF, sunflower; THC, tetrahydrocannabinol; THCA, tetrahydrocannabinolic acid.

a RL was 0.5 mg/mL.

b Twenty-one terpenes were specifically measured: alpha pinene, beta pinene, myrcene, limonene, terpinolene, linalool, terpineol, caryophyllene, humulene, 3-carene, cis-ocimene, eucalyptol, trans-ocimene, fenchol, borneol, valencene, cis-nerolidol, trans-nerolidol, guaiol, alpha-bisabolol, sabinene. RL was 0.01%.

c *Nine terpenes were specifically measured: alpha-pinene, beta-pinene, myrcene, limonene, terpinolene, linalool, terpineol, caryophyllene, humulene. RL was 0.01%*.

### Subject Selection

The purpose-bred animals were acquired from a colony at VivoCore Inc. (Fergus, ON, Canada). The inclusion criteria for the study were good general health as determined by the veterinarian; stable weight over a 10-day period preceding study start and a weight of 9–15 kg; and, under 8 years of age. Exclusion criteria were pregnant or lactating dogs; use of medications or supplements during the course of the study; receipt of cannabinoid or cancer-related therapies in 2 months preceding study start; receipt of any test substance within a month prior to study start; existing or a history of cancer, blood- or immunology-related disease or other chronic morbidity (including open wounds, psoriatic or allergic skin conditions, chronic diarrhea, chronic oral gum or tooth disease, or cardiovascular disease).

### Animal Care

The dogs were individually housed in stainless steel metabolic cages (height, width, depth = 75 × 90 × 102 cm) and those in the same treatment group were exercised together. Environmental controls for the animal housing area were electronically set to maintain a temperature of 18.7–26.2°C and a 12-h light/dark cycle. Animals were fed a standard commercial dry canine diet in stainless steel bowls (Purina ProPlan Savor Adult—Chicken and Rice Formula) once daily, 7–10 h after dosing. Food was left for 1 h and the food quantity offered (1.25–2.75 cups) was based on body weight. Water was available *ad libitum* in stainless steel bowls. Upon study completion, animals were returned to their colony at VivoCore Inc.

### Measurable Outcomes

Throughout the study period, food consumption and 24-h activity (Mini-Mitter® Actiwatch-64® Mini-Mitter Co., Inc., Bend, OR) were measured daily and animal health observations occurred twice daily. Body weights were collected throughout the acclimation period, once during the study period, and once upon study completion. Following administration of the cannabinoid or placebo oils, animals were monitored up to 9 h post-dose for body temperature (measured rectally), respiration rate (by observation), and heart rate (stethoscope). Observations of the animals were also conducted every 1–3 h post-dosing through the first 9 h and then at 12 and 24 h. Subjects were observed for any signs that would not be expected in normal dogs and for the occurrence of AEs. Experienced veterinary technicians and/or a licensed veterinarian conducted the clinical observations and physical assessments.

AEs were rated for severity as (i) mild—activities of daily living (ADL) not impacted and no intervention indicated; (ii) moderate—ADL moderately limited (non-invasive intervention may be indicated); or (iii) severe/medically significant—ADL significantly limited (23). If one dog in a treatment group experienced a severe/medically significant AE, no further treatments were administered to that dog. If two dogs in the same treatment group experienced severe/medically significant AEs, subsequent treatments ceased for all dogs in that group.

### Blood Collections and Analyses

For analysis of Complete Blood Count (CBC) and clinical chemistry, 4 mL of blood was drawn by direct venipuncture from a cephalic or jugular vein; 2 mL was placed into an evacuated serum separator tube (SST) and another 2 mL into an evacuated K_2_EDTA tube. These blood collections occurred during acclimation (baseline), mid-study (after five doses of placebo oil, and three doses of cannabinoid oils), and 7 days following the final dose of the cannabinoid or placebo oils. Additionally, they occurred 24 h following the final dose of the cannabinoid oils. With the occurrence of a severe AE, blood was drawn immediately, and 24 h and 7 days thereafter.

For analysis of CBD, THC, and their metabolites (7-COOH-CBD and 11-OH-THC), 2 mL of blood was drawn and placed into an evacuated K_2_EDTA tube. These blood collections occurred before and after the ninth dose of the CBD oil and THC oil (i.e., pre-dose, and at 1, 2, 4, 6, and 24 h post-dose). These blood collections also occurred 7 days following the final dose of the cannabinoid or placebo oils.

Blood in the evacuated SSTs was allowed to clot for a minimum of 30 min but no more than 1 h following collection, then was centrifuged at 1,525–1,992 relative centrifugal force (rcf) at 20°C for 10 min. K_2_EDTA tubes were centrifuged at 2,800–3,000 revolutions per minute (rpm) for 10 min at 4°C. The SSTs and K_2_EDTA tubes were stored at 2 to 8°C and on the same day as blood collection were transported to Antech Diagnostics (Mississauga, ON) for analysis (CBC and clinical chemistry) or further processing. For the K_2_EDTA tubes intended for analysis of CBD, THC, and their metabolites, plasma was separated into two equal aliquots and stored at −80°C until shipment on dry ice to the bioanalytical laboratory for analysis (InterVivo Solutions, Inc., Mississauga, ON).

CBD, THC, 7-COOH-CBD, and 11-OH-THC were analyzed by liquid chromatography tandem mass spectrometry (LC-MS/MS) (QTRAP® 6500 with an Exion LC™ system, AB Sciex LP). The analytes CBD, THC and 11-OH-THC were purchased as analytical reference solutions from Sigma-Aldrich; 7-COOH-CBD was purchased from Toronto Research Chemicals. The analytes were chromatographically separated on a Phenomenex Kinetex Phenyl-Hexyl column (2.1 × 50 mm, 2.6 μm) using gradient elution (mobile phase A = 0.1% formic acid in water and mobile phase B = 0.1% formic acid in acetonitrile) at a flow rate of 0.4 mL/min. The mass spectrometer was operated in multiple reaction monitoring (MRM) mode with a turbo ion spray interface. Internal standards were deuterated analogs of THC, CBD, and 11-OH-THC (THC-d_3_, CBD-d_3_, 11-OH-THC-d3, Toronto Research Chemicals); paclitaxel was used as the internal standard for 7-COOH-CBD. All four compounds were analyzed in one run.

Calibration standards were prepared in blank pooled dog plasma (with K_2_EDTA as anticoagulant). Ten calibration standards over the range of 0.25–2,000 ng/mL (CBD, THC) or 0.5–2,000 ng/mL (7-COOH-CBD, 11-OH-THC) were used and included a blank sample (without internal standard) and a zero sample (with internal standard). Plasma standards and samples were extracted by the addition of a solution of 50/50 methanol/acetonitrile containing the internal standards to precipitate the proteins. A sample batch consisted of the following: 3 replicates of a system suitability standard (containing the analyte and internal standard), calibration standards in ascending order including a blank sample (without internal standard), a zero sample (with internal standard), and 10 non-zero standards, the assay samples, followed by the 3 replicates of the system suitability sample. Calibration standards bracketed an analysis batch of >40 samples.

Acceptance criteria for method qualification and sample analysis were: (1) that at least 75% of the non-zero calibration standards be included in the calibration curve with all back-calculated concentrations within ±20% deviation from nominal concentrations (except for the lower level of quantification, LLOQ, where ±25% deviation was acceptable), (2) the correlation coefficient (r) of the calibration curve must be ≥0.99, and (3) the area ratio variation between the pre- and post-run injections of the system suitability samples is within ±25%.

### Data Analysis

Measures of central tendency (mean), variability (standard deviation, standard error of mean), and all figures were generated by GraphPad Prism version 8.1.2 for Windows, GraphPad Software, San Diego, California, USA, www.graphpad.com.

## Results

Four of 24 dogs evaluated for inclusion into the study were excluded from study enrollment following the acclimation period due to recurring loose stool (*n* = 1), recurring ear infections (*n* = 1), and inadequate maintenance of body weight (*n* = 2). Therefore, 20 dogs were randomized to treatment groups (with 4 dogs per group). The mean (SD) body weights of each group at baseline were as follows: MCT oil: 12.2 kg (1.1 kg); CBD oil: 10.3 kg (0.4 kg); THC oil: 12.3 kg (1.0 kg); SF oil: 11.9 kg (1.5 kg); CBD/THC oil: 11.3 kg (0.9 kg).

Of the cannabinoid oils tested, dosing of the CBD oil had the least effect on food intake and physical activity. Specifically, food intake decreased on dosing days as compared to non-dosing days by 4.6% (MCT oil), 8.1% (CBD oil), 27.9% (THC oil), and 44.7% (CBD/THC oil), with no changes observed between these periods with SF oil (data not shown). Dogs were not exercised/socialized on dosing days and physical activity (measured by Actiwatch-64®) was thus reduced across all groups on dosing days as compared to non-dosing days by 12.9% (MCT oil), 19.2% (CBD oil), 19.8% (THC oil), 24.4% (SF oil), and 40.7% (CBD/THC oil) (data not shown). Despite these changes, body weights remained stable throughout the study period across the five groups.

### Dose Escalation and Subject Discontinuation

The two placebo oils were tested up to 10 escalating volumes while the CBD oil, THC oil, and CBD/THC oil were tested up to the tenth, tenth, and fifth dose, respectively; titration to maximum doses of 640.5 mg CBD (~62 mg/kg), 597.6 mg THC (~49 mg/kg), and 140.8/96.6 mg CBD/THC (~12 mg/kg CBD + 8 mg/kg THC), respectively, was thus achieved (Tables 1, 2). For the cannabinoid oils, the second cannabinoid dose was 2- to 2.5-fold greater than the first dose; thereafter, serial doses increased by 1.2- to 2-fold.

One of four dogs in the THC oil group experienced severe ataxia at the 7th dose and was discontinued from further dosing. Two of four dogs in the CBD/THC oil group experienced severe ataxia and/or lethargy at the fourth or fifth doses (one dog at the fourth dose and a second dog at the fifth dose) and thus further dosing ceased for all dogs in this group. No dogs were discontinued from the CBD oil or placebo oil groups as a result of AEs.

### Adverse Events

**Figure 1:**
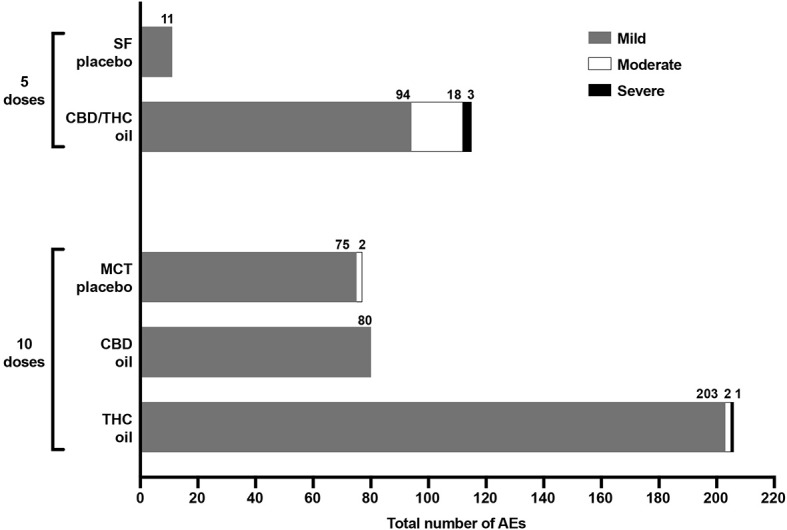
Total number and severity (mild, moderate, severe) of AEs experienced across five escalating doses of SF oil placebo (*n* = 4) and CBD/THC oil (*n* = 3 or 4) and 10 escalating doses of MCT oil placebo (*n* = 4), CBD oil (*n* = 4), and THC oil (*n* = 3 or 4). There were no moderate or severe AEs experienced with CBD oil and SF oil.

AEs were reported in all 20 dogs across the five groups. Of the total number of AEs observed across the entire study (*n* = 505), 104 AEs occurred in the placebo groups across 10 escalating volumes (77 AEs with MCT oil and 27 AEs with SF oil), and 401 AEs occurred across the three cannabinoid groups: 80 AEs with CBD oil (10 doses), 206 AEs with THC oil (10 doses), and 115 AEs with CBD/THC oil (five doses) (Figure 1). The SF oil group was the placebo control for the CBD/THC oil group, the latter which was delivered up to five doses; as such, while there were 27 AEs across 10 doses of SF oil, there were 11 AEs across the first five doses of SF oil (Figure 1).

**Figure 2:**
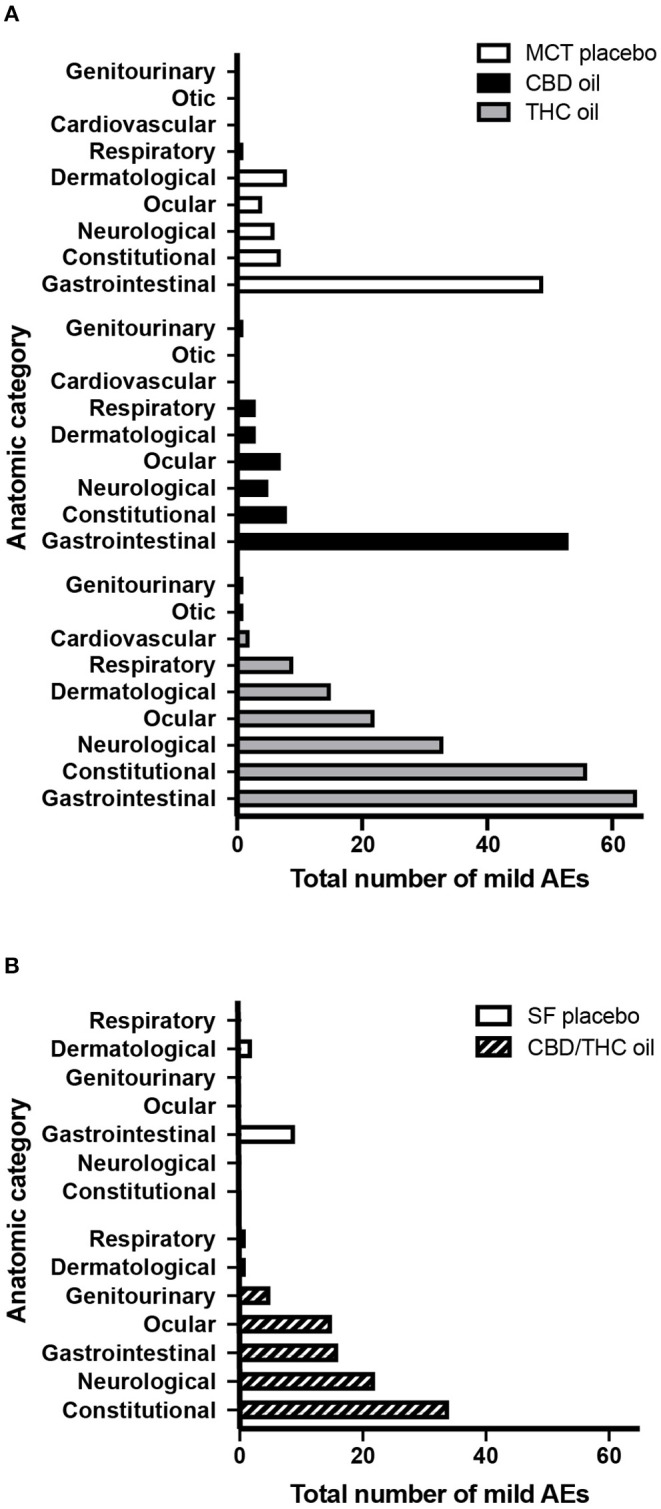
Total number of mild AEs per anatomic category. Mild AEs accounted for the majority (94.9%) of AEs. **(A)** Mild AEs across 10 doses of MCT oil placebo (*n* = 4), CBD oil (*n* = 4), or THC oil (*n* = 3 or 4). **(B)** Mild AEs across five doses of SF oil placebo (*n* = 4) or CBD/THC oil (*n* = 3 or 4). Gastrointestinal = nausea, vomiting, diarrhea, or other (hematemesis or blood or mucus in stool); Constitutional = lethargy, hyperesthesia, hypothermia, or other (weight loss, hypertonia, eyebrows raised and no blinking, abnormal posture, vocalization); Neurological = tremor (including hiccups) or ataxia; Ocular = mydriasis or other (epiphora, conjunctivitis, blepharospasm); Dermatological = pruritus or other (skin ulceration, purpura, alopecia, erythema, granuloma); Respiratory = nasal discharge or bradypnoea; Cardiovascular = bradycardia; Otic = external ear inflammation; Genitourinary = urinary incontinence or hematuria.

**Figure 3:**
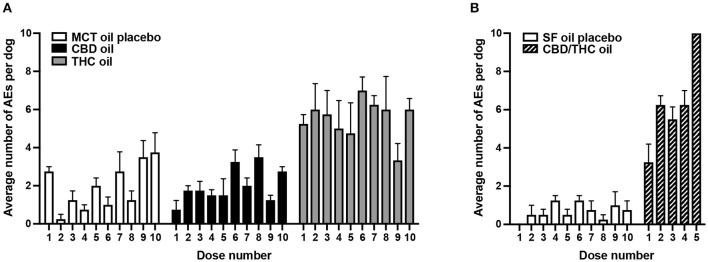
Average number (SEM) of AEs per dog per administered dose of oil. **(A)** Ten doses of MCT oil placebo (*n* = 4), CBD oil (*n* = 4), or THC oil (*n* = 3 or 4) were administered. **(B)** Ten doses of SF oil placebo (*n* = 4) and five doses of CBD/THC oil (*n* = 3 or 4) were administered.

Across the three groups receiving cannabinoid oils, the fewest AEs were reported in the CBD oil group (across 10 doses) as compared to the THC oil group (across 10 doses) and the CBD/THC oil group (across five doses) (Figure 1). Compared to dogs receiving the CBD oil, dogs receiving the THC oil experienced 2.6-fold more total AEs (Figure 1) and, more specifically, 7-fold more neurological and constitutional AEs, 5-fold more dermatological AEs, and 3-fold more ocular and respiratory AEs (Figure 2). The greatest difference between the CBD oil and THC oil groups with respect to the occurrence of AEs occurred at the first dose at which point there was a 7-fold difference in the average number of AEs experienced per dog (Figure 3). For the remaining nine doses, dogs receiving the THC oil experienced between a 2- and 3-fold greater number of AEs per dose vs. dogs receiving the CBD oil (Figure 3). With respect to the CBD/THC oil group, there was a steep increase in the average number of AEs per dog at the fifth dose; each of the three dogs experienced 10 AEs at this dose vs. the other two cannabinoid oil groups wherein an average of 4.8 AEs (THC oil group) and 1.5 AEs (CBD oil group) were experienced per dog (Figure 3). It is noteworthy that the total number of AEs and the AE profile in the CBD oil group were comparable to the MCT placebo oil group (Figures 1, 2). Moreover, across the cannabinoid oil groups, at each escalating dose, dogs in the CBD oil group experienced a lower average number of AEs as compared to the THC oil group and the CBD/THC oil group (Figure 3).

The majority of AEs in each of the five groups, and collectively across all five groups, were mild. Mild AEs accounted for 479 of the 505 total study AEs (94.9%) (Figure 1). Mild AEs occurred in all subjects and mainly manifested as gastrointestinal (nausea, emesis, diarrhea), constitutional (lethargy, hyperesthesia), or neurological (muscle tremor, ataxia) symptoms (Figure 2). The proportion of mild AEs that were gastrointestinal in each group was: 49/75 (65.3%) (MCT oil; 10 doses), 53/80 (66.3%) (CBD oil; 10 doses), 64/203 (31.5%) (THC oil; 10 doses), 9/11 (81.8%) (SF oil; five doses), 16/94 (17.0%) (CBD/THC oil; five doses). The proportion of mild AEs that were constitutional or neurological in each group was: 13/75 (17.3%) (MCT oil; 10 doses), 13/80 (16.3%) (CBD oil; 10 doses), 89/203 (43.8%) (THC oil; 10 doses), 0/11 (SF oil; five doses), 56/94 (59.6%) (CBD/THC oil; five doses). Thus, across the two placebo groups (SF oil, MCT oil) and the CBD oil group, a greater proportion of mild AEs were gastrointestinal vs. constitutional/neurological whereas in the THC oil and CBD/THC oil groups, a greater proportion of mild AEs were constitutional/neurological vs. gastrointestinal.

**Figure 4:**
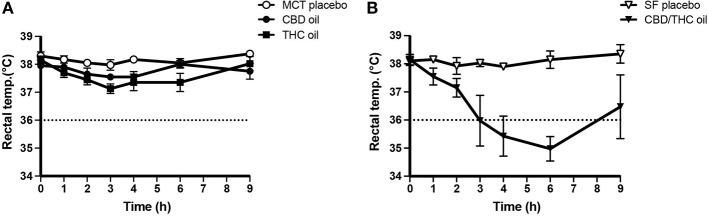
Rectal temperature (mean ± SEM) after the 4th dose of the cannabinoid oils or the 6th dose of the placebo oils, measured on the same calendar day. **(A)** MCT oil (*n* = 4) vs. CBD oil (*n* = 4; 137.3 mg CBD) vs. THC oil (*n* = 4; 109.6 mg THC). **(B)** SF oil (*n* = 4) vs. CBD/THC oil (*n* = 4; 105.6/72.5 mg CBD/THC). Rectal temperature was measured pre-dose (time 0) and 1, 2, 3, 4, 6, and 9 h post-dose. The dotted line corresponds to a rectal temperature of 36.0°C, below which the dogs were considered to have hypothermia.

There were no moderate AEs in the CBD oil group at any of the doses tested. Moderate AEs accounted for 22 of the 505 total study AEs (4.4%) and occurred in 40% of the subjects (8 of 20 dogs) across three groups: MCT oil (two dogs at the tenth dose), THC oil (two dogs at the third or seventh doses), or CBD/THC oil (four dogs across the five doses tested) (Figure 1). Moderate AEs manifested as constitutional (lethargy, hypothermia) or neurological (ataxia) symptoms. The most common moderate AE was hypothermia (rectal temperature <36.0°C), which accounted for 14 of the 22 moderate AEs (64%). The majority of hypothermia occurrences (13 of 14) occurred in the CBD/THC oil group, wherein all 4 dogs experienced hypothermia at four of the five doses tested (excluding the first dose). The lowest dose of CBD/THC oil at which hypothermia occurred was at the second dose (35.2/24.2 mg CBD/THC = ~3 mg/kg CBD and ~2 mg/kg THC). The remaining hypothermia event occurred in the THC oil group at the third dose (82.2 mg THC = ~7 mg/kg THC). Indeed, as compared to the other cannabinoid and placebo oils, a greater decline in the rectal temperature of dogs occurred following intake of the CBD/THC oil (Figure 4). Moderate AEs were transient and resolved in 3–24 h. Importantly, there were no moderate AEs, including hypothermia, in the CBD oil group at any of the doses tested.

There were no severe/medically significant AEs in the CBD oil group at any of the doses tested. Severe AEs accounted for 4 of the 505 total study AEs (0.8%) (Figure 1) and occurred in 15% of the subjects (3 of 20 dogs) across two groups: THC oil (one dog at the seventh dose) and CBD/THC oil (one dog at the fourth dose and a second dog at the fifth dose). Severe AEs manifested as severe ataxia and/or lethargy and were transient, resolving in 9–28 h. Plasma levels of CBD, THC, and their metabolites measured upon observation of severe AEs were 785 ng/mL THC and 93.9 ng/mL 11-OH-THC (THC oil group) and 133–296 ng/mL CBD and 99.5–361 ng/mL THC (CBD/THC oil group). Bloodwork results from animals experiencing severe AEs revealed abnormalities (low platelet count, high pancreatic sensitive lipase, high white blood cell count, high neutrophils, and/or high monocytes) only in the two dogs administered the CBD/THC oil; these abnormalities resolved within 24 h. There were no abnormal physical findings of the dogs on final examinations conducted by the facility's veterinarian.

### Hematological Changes

#### Clinical Chemistry and Complete Blood Count

For the cannabinoid oil groups, blood was collected at baseline, and 24 h and 7 days after the final dose. Overall, hematological parameters were generally normal for the dogs across these groups at 24 h and 7 days after the final dose, with a few exceptions as outlined below.

With respect to changes in clinical chemistry parameters suggestive of altered liver function, while possibly not clinically pathologic, we interpreted a notable change to be: (i) at least a 2-fold increase from baseline to post final dose timepoints (24 h or 7 days) in total bilirubin or plasma levels of liver enzymes [alkaline phosphatase (ALP), alanine aminotransferase (ALT), aspartate aminotransferase (AST), gamma-glutamyl transpeptidase (GGTP)]; and (ii) the post final dose level (24 h or 7 days) in the above parameters reached or exceeded the upper limit of normal. Applying these criteria, one dog in the CBD oil group and one dog in the CBD/THC oil group experienced 2.9-fold or 3.6-fold increases in ALP from baseline to 24 h following the last administered dose, which was the tenth dose (CBD oil) or the fifth dose (CBD/THC oil), respectively (Table 4). Moreover, the post final dose level (at 24 h) approached or exceeded the upper cut-off of normal for ALP. Comparing the 7 days post final dose ALP levels for these two dogs to the 24-h post final dose levels showed a downward trend (Table 4). Plasma levels of liver enzymes and total bilirubin were stable in the THC oil group.

**Table 4:** Clinical chemistry plasma parameters indicative of liver function as measured in healthy Beagle dogs administered cannabinoid oils.

	**AST (U/L)**			**ALT (U/L)**			**ALP (U/L)**			**GGTP (U/L)**			**Total Bilirubin (μmol/L)**		
	**RR** **=** **15–66 U/L**			**RR** **=** **12–118 U/L**			**RR** **=** **5–131 U/L**			**RR** **=** **1–12 U/L**			**RR** **=** **0.0–5.1** **μmol/L**		
	**BSN**	**24 h post FD**	**7 d post FD**	**BSN**	**24 h post FD**	**7 d post FD**	**BSN**	**24 h post FD**	**7 d post FD**	**BSN**	**24 h post FD**	**7 d post FD**	**BSN**	**24 h post FD**	**7 d post FD**
**CBD OIL**															
Dog 1	23	21	21	21	21	20	44	127^a^	93	3	3	4	2.4	1.6	1.3
Dog 2	24	23	23	23	25	24	80	94	123	3	3	3	3.2	2.2	2.3
Dog 3	21	17	18	29	28	29	43	69	66	6	4	4	2.3	1.7	1.7
Dog 4	25	23	22	24	45	31	48	83	80	4	6	4	2.2	1.1	1.5
Mean (SD)	23.3 (1.7)	21.0 (2.8)	21.0 (2.2)	24.3 (3.4)	29.8 (10.6)	26.0 (5.0)	53.8 (17.6)	93.3 (24.7)	90.5 (24.3)	4.0 (1.4)	4.0 (1.4)	3.8 (0.5)	2.5 (0.5)	1.7 (0.5)	1.7 (0.4)
**THC OIL**															
Dog 1	16	15	13	19	22	20	40	46	49	5	3	3	1.8	1.6	1.4
Dog 2^b^	23	21	19	21	24	20	26	37	27	4	1	5	2.6	2.1	1.8
Dog 3	16	23	18	19	21	23	21	27	29	2	4	4	2.8	2.0	1.7
Dog 4	26	19	20	22	24	23	35	33	39	4	4	2	2.2	1.8	1.8
Mean (SD)	20.3 (5.1)	19.0 (4.0)	17.0 (3.6)	20.3 (1.5)	22.3 (1.5)	22.0 (1.7)	30.5 (8.6)	35.3 (9.7)	39.0 (10.0)	3.8 (1.3)	3.7 (0.6)	3.0 (1.0)	2.4 (0.4)	1.8 (0.2)	1.6 (0.2)
**CBD/THC OIL**															
Dog 1	27	18	19	40	42	41	52	189^c^	88	3	3	4	2.5	1.7	2.0
Dog 2^d^	24	21	24	30	32	34	60	96	79	3	3	4	2.0	2.6	1.7
Dog 3	26	24	19	28	24	39	36	55	42	2	3	1	3.7	1.9	2.3
Dog 4	21	19	16	21	66	29	25	41	37	3	5	5	2.6	2.3	2.1
Mean (SD)	24.5 (2.6)	20.3 (3.2)	18.0 (1.7)	29.8 (7.8)	44.0 (21.1)	36.3 (6.4)	43.3 (15.7)	95.0 (81.7)	55.7 (28.1)	2.8 (0.5)	3.7 (1.2)	3.3 (2.1)	2.7 (0.7)	2.0 (0.3)	2.1 (0.2)

ALP, alkaline phosphatase; ALT, alanine aminotransferase; AST, aspartate aminotransferase; BSN, baseline; CBD, cannabidiol; d, days; FD, final dose; GGTP, gamma-glutamyl transpeptidase; h, hours; RR, reference range; SD, standard deviation; THC, delta-9-tetrahydrocannabinol; U/L, units per liter.

a ALP level 2.9-fold higher than baseline and approaching the upper limit of normal (131 U/L).

b The 7th dose was the last dose due to a severe/medically significant AE with this dose. Calculation of the mean (SD) for all post FD outcomes excluded this dog's data.

c ALP level 3.6-fold higher than baseline and exceeded the upper limit of normal (131 U/L).

d *The 4th dose was the last dose due to a severe/medically significant AE with this dose. Calculation of the mean (SD) for all post FD outcomes excluded this dog's data. For the remaining dogs in this group, the 5th dose was the last dose*.

With respect to the remaining CBC and clinical chemistry parameters, blood collected 24 h following the final dose of the cannabinoid oils showed only a few abnormalities as based on laboratory reference ranges. These abnormalities occurred in 1 or 2 dogs in the cannabinoid oil groups and consisted of low creatine phosphokinase (1 dog in CBD oil group); low amylase, high sodium, or high pancreatic sensitive lipase (2 dogs in THC oil group); high pancreatic sensitive lipase (1 dog in the CBD/THC oil group) (data not shown). All abnormalities resolved 7 days following the final dose.

#### Cannabinoids and Their Metabolites

**Figure 5:**
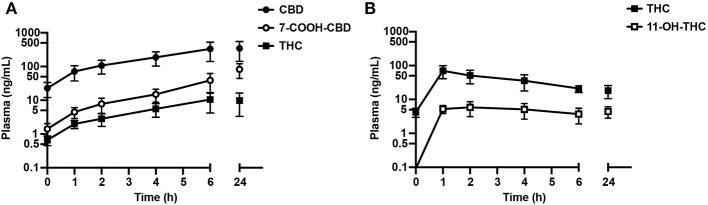
Plasma levels (mean ± SEM) of CBD, THC, and their metabolites (7-COOH-CBD, 11-OH-THC) pre-dose, and at 1, 2, 4, 6, and 24 h following the 9th dose of **(A)** CBD oil (549.0 mg CBD; *n* = 4) or **(B)** THC oil (448.2 mg THC; *n* = 3). Following exposure to the CBD oil, levels of 11-OH-THC were not detected or were below the lower level of quantitation in the majority of dogs. Following exposure to the THC oil, there were no detectable levels of CBD or its metabolite (7-COOH-CBD).

Following intake of the ninth dose of the CBD oil (*n* = 4) or THC oil (*n* = 3), plasma levels of CBD, THC, and their metabolites (7-COOH-CBD, 11-OH-THC) were measured 1, 2, 4, 6, and 24 h post-dose; levels of these parameters were also measured pre-dose (Figure 5).

Intake of the ninth dose of the CBD oil (549.0 mg CBD; ~53 mg/kg) led to steady inclines in plasma CBD, 7-COOH-CBD and THC over the 6-h post-dose period (Figure 5). Levels (mean/SEM) of CBD and THC at 6 h post-dose were similar to levels 24 h post-dose [CBD: 334.0 ± 193.0 ng/mL (6 h) and 347.0 ± 204.5 ng/mL (24 h); THC: 10.5 ± 6.3 ng/mL (6 h) and 9.8 ± 6.5 ng/mL (24 h)]. In contrast, levels of 7-COOH-CBD were 2-fold lower at 6 vs. 24 h post-dose (39.1 ± 23.0 ng/mL and 82.8 ± 38.4 ng/mL, respectively).

Following intake of the ninth dose of the THC oil (448.2 mg THC; ~37 mg/kg), mean (SEM) plasma THC and 11-OH-THC reached maximum levels of 69.8 ± 28.8 ng/mL (THC, 1 h post-dose) and 5.9 ± 2.7 ng/mL (11-OH-THC, 2 h post-dose), respectively, with steady declines observed thereafter over the 6-h post-dose period. Mean plasma THC at 6 h post-dose (21.1 ± 5.1 ng/mL) was similar to its level 24 h post-dose (18.2 ± 7.6 ng/mL). Mean plasma 11-OH-THC at 6 h post-dose (3.7 ± 1.8 ng/mL) was similar to its level 24 h post-dose (4.5 ± 1.7 ng/mL).

Across the cannabinoid oil groups, cannabinoids and their metabolites were measured 7 days following the last administered dose. Seven days following intake of the last (tenth) dose of the CBD oil (640.5 mg CBD; ~62 mg/kg; *n* = 4), CBD was detected in all four dogs (3.6–31.7 ng/mL) while levels of 7-COOH-CBD were detected in half the dogs (1.4–1.8 ng/mL) (data not shown). Seven days following intake of the last (tenth) dose of the THC oil (597.6 mg THC; ~49 mg/kg; *n* = 3), THC was detected in all three dogs (2.8–8.2 ng/mL), while 11-OH-THC was not detected in any dogs (data not shown). Seven days following the intake of the last (fifth) dose of the CBD/THC oil (140.8/96.6 mg CBD/THC; ~12 mg/kg CBD +8 mg/kg THC; *n* = 3), CBD and THC were detected in all three dogs (CBD: 8.4–18.8 ng/mL; THC: 6.2–12.2 ng/mL), while 7-COOH-CBD was detected in one of three dogs (1.4 ng/mL) and 11-OH-THC was not detected in any of the three dogs (data not shown).

## Discussion

In our study, a CBD-predominant oil consumed via 10 escalating doses containing 18.3–640.5 mg CBD per dose (~2–62 mg/kg) led to only mild AEs and no moderate or severe AEs. Importantly, the number and type of AEs that occurred in the CBD oil group were comparable to the corresponding placebo group (MCT oil). In both groups, the majority of AEs were gastrointestinal, which could have been due to discomfort with oral gavage, oil volume, and/or the MCT oil carrier.

The safety and tolerability of CBD, as determined primarily in rodents and humans, has been extensively reviewed (6–8). In humans, oral doses of CBD ranging from a minimum of 15 or 20 mg/day (24, 25) to a maximum of 1,200–1,500 mg/day (26–28) have been well-tolerated, with no significant side effects. In mice, oral doses ranging from 3 to 100 mg/kg showed no significant effects on catalepsy (29). In dogs, CBD has shown to be well-tolerated at doses ranging from 2 to 2.5 mg/kg twice daily for 4 or 12 weeks (17, 21) to 5 or 10 mg/kg twice daily for 6 weeks (20). Indeed, in their critical review report on CBD, the World Health Organization (WHO) concluded that “CBD is generally well-tolerated with a good safety profile (…) and relatively low toxicity” (8).

Previous studies have reported increases in liver enzymes, specifically ALP, in dogs receiving CBD orally at doses ranging from 2 mg/kg (twice daily) to 10 mg/kg (twice daily) for 4, 6, or 12 weeks (17, 20, 21) with increases observed as early as 2 weeks following treatment initiation (20). In our study, one dog in the CBD oil group and one dog in the CBD/THC oil group experienced 2.9- or 3.6-fold increases in ALP from baseline to 24 h following the tenth dose (CBD oil) or fifth dose (CBD/THC oil). These changes were not considered clinically significant by the veterinarian providing oversight for the study. Elevated liver enzymes with exposure to cannabis or CBD have also been observed in humans and rodents. Cross-sectional human studies have shown plasma ALP to be elevated in habitual daily cannabis users (30, 31) with hepatomegaly also observed (31). Increases in plasma AST, ALT, and liver-to-body weight ratios were observed in rodents treated with CBD (oral gavage; 615 mg/kg for 10 days) albeit there were unremarkable changes in liver enzymes in lower dose groups (61.5 and 184.5 mg/kg) (32). The potential short- and long-term effects of CBD on liver function in dogs warrant further investigation.

Only in the CBD/THC oil group were severe AEs experienced by more than one dog causing cessation of dosing after the fifth dose. Researchers have acknowledged that CBD can have interactions with THC (33) and that CBD is not always a functional antagonist of THC (34). Indeed, there is evidence in rodents (33–37) and humans (38) that CBD can potentiate, rather than antagonize, the psychoactive and physiological effects of THC (e.g., locomotor activity suppression, hypothermia, hypoactivity). The interaction between these two exogeneous phytocannabinoids may be pharmacokinetic (CBD modifies the effect of THC through changes in absorption, distribution, and/or elimination) or pharmacodynamic (CBD modifies the effect of THC via additive, synergistic, or antagonistic effects). The interaction depends on whether CBD is administered prior to THC (pharmacokinetic interaction more likely) or concurrently with THC (pharmacodynamic interaction more likely) (7, 34, 39) and also on the dose ratio of the compounds (39, 40). Regarding the latter, when CBD/THC are simultaneously co-administered at a mean (±standard deviation) dose ratio of 8.1 (±11.1), antagonistic effects of CBD on THC have been observed (a pharmacodynamic interaction) whereas at a ratio of 1.8 (±1.4), CBD has been shown to potentiate the effects of THC (a pharmacokinetic interaction) (39, 41). Our CBD/THC oil had a CBD/THC dose ratio of 1.5. It therefore follows that the effects of THC may have been potentiated by CBD via a pharmacokinetic interaction. CBD is known to be a potent inhibitor of hepatic drug metabolism (39) by inactivating cytochrome P450 enzymes. When co-administered with THC, this effect can delay the metabolism of THC in the liver (7, 34). Notwithstanding that the levels of other cannabinoids (THCA, CBDA, CBG, CBGA, CBN, CBC) and terpenes in the CBD/THC oil fell below reporting limits (0.5 mg/mL for cannabinoids and 0.01% for terpenes), their potential interaction with CBD and/or THC in the oil and their contribution to the overall effect of the CBD/THC oil cannot be precluded (this synergy is often called the “entourage effect”).

Our data show a clear distinction in AEs associated with a CBD-predominant oil vs. oils containing higher levels of THC in that constitutional (lethargy, hyperesthesia, hypothermia) and neurological (tremor, ataxia) AEs most commonly occurred in dogs receiving the THC oil or the CBD/THC oil. Suppression of locomotor activity (hypolocomotion), catalepsy, and hypothermia have been observed across species (dogs, cats, rodents, chickens) and associated with exposure to cannabis, THC, or THC and CBD in combination, but not CBD alone (33, 37, 42–45). Animal studies have shown that the production of these effects is dependent on cannabinoid type 1 (CB1) receptor activation (45). CB1 receptors are located primarily in central and peripheral neurons and are found in the highest densities in the neuron terminals of the basal ganglia, cerebellum, hippocampus neocortex, hypothalamus, and limbic cortex—areas which, among other functions, are involved in motor activity, coordination, and sedation (46, 47). THC, a CB1 receptor partial agonist, has a high affinity for CB1 receptors (48) and is capable of binding and activating them at orthosteric sites (45, 49). In contrast, CBD does not bind to orthosteric sites of these receptors like THC (49). In our study, there was a lower proportion of constitutional and neurological AEs following intake of CBD oil (vs. THC oil and CBD/THC oil) and no moderate or severe AEs were experienced by dogs in the CBD oil group. CBD's lack of interaction with orthosteric sites of CB1 receptors (49) is a plausible explanation for the fewer and less severe AEs experienced by dogs receiving CBD oil.

Plasma levels of CBD, THC, and their metabolites were highly variable between dogs in the same treatment group receiving either the ninth dose of CBD oil or THC oil. Others have also observed high variability in cannabinoid (CBD, THC) blood concentrations across dogs in single dose pharmacokinetic studies (16, 19) and repeated administration studies (21), which may be explained by differences in absorption rates or cannabinoid metabolism across subjects. Following the ninth dose of the CBD oil (~53 mg/kg), maximum plasma CBD levels achieved across the four dogs in the CBD oil group (62.3–896.0 ng/mL, at 4, 6, or 24 h post-dose) were comparable to the range in plasma CBD levels achieved across nine dogs (~130–940 ng/mL) following repeated daily CBD dosing (2.5 mg/kg twice daily) for 12 weeks (21). With respect to plasma levels reached following the ninth dose of the THC oil (~37 mg/kg), at 24 h post-dose, mean plasma THC (18.2 ng/mL) and 11-OH-THC (4.5 ng/mL) across three dogs approximated mean plasma levels reached by eight dogs receiving a cannabis extract (2.7 mg/kg THC + 2.5 mg/kg CBD) for 56 weeks: 22.0 ng/mL (THC) and 6.7 ng/mL (11-OH-THC) (11). Thus, it appears that repeated daily dosing of lower cannabinoid doses can achieve comparable plasma CBD or THC levels to acutely administered higher doses.

Contrary to earlier assertions that CBD has low bioavailability after oral administration to animals, including dogs (18, 50), our study showed circulating plasma CBD and 7-COOH-CBD in all dogs receiving the ninth dose of CBD oil at all post-dose timepoints (1, 2, 4, 6, and 24 h). Based on these results, it appears that a first pass effect through the liver did not eliminate the systemic availability of CBD following its oral ingestion. Given the highly lipophilic nature of CBD (50), its administration in a lipid solvent (MCT oil) in the present study may have increased its bioavailability. Zgair et al. (51) showed that co-administration of lipids with oral CBD increased systematic availability of CBD by almost 3-fold in rats as compared to lipid-free formulations. Overnight fasting of the dogs in the present study prior to dosing may have also improved bioavailability. Lebkowska-Wieruszewska et al. (19) showed improved cannabinoid (THC) bioavailability in fasted vs. fed dogs, with a lower T_max_ and higher C_max_ achieved for THC in the fasted condition. The approximate cumulative CBD dose administration from the first to ninth dose was 2122.9 mg. Detected plasma levels of CBD may also be reflective of CBD accumulation in plasma with dose escalation over time.

Fasted dogs receiving the ninth dose of the THC oil (cumulative THC from first to ninth dose = 1653.5 mg) achieved maximum THC plasma levels at 1-h post-dose. Related findings are those reported by Lebkowska-Wieruszewska et al. (19) who calculated median T_max_ levels for plasma THC of 1.25 h in fasted dogs orally dosed with Bedrocan® (1.5 mg THC/kg). It is reported in the literature that following oral ingestion of cannabis, maximum plasma THC levels are reached within 1–2 h (52). Decreases in plasma THC observed in our study after 1 h are suggestive of the uptake of THC by fat tissues and highly vascularized tissues, such as the brain and muscle (52). Indeed, in the THC oil group, the average onset of neurological AEs (ataxia, tremor) or constitutional AEs (lethargy, hyperesthesia, hypothermia, hypertonia) was ~4 h post-dose (range of onset was 1–24 h).

Both CBD and THC were detected in plasma 1 week following administration of the final dose of either CBD oil (~62 mg/kg; *n* = 4) or THC oil (~49 mg/kg; *n* = 3), at levels ranging from 3.6 to 31.7 ng/mL CBD (CBD oil) and 2.8 to 4.6 ng/mL THC (THC oil). This finding is relevant to future studies which apply a crossover design and include a washout period. That quantifiable levels of CBD were observed one week following dose exposure is also interesting given that CBD has been reported to have a relatively rapid elimination in dogs [CBD has been shown to be bio-transformed in dogs via hydroxylation, carboxylation, and conjugation (10, 18)].

The cannabinoid oils used in this study are proprietary formulations with relatively high concentrations of CBD and/or THC and low concentrations of other cannabinoids and terpenes. Given the multitude of factors that can affect the proportion of constituents in the cannabis plant (light, temperature, humidity, soil type during cultivation, plant genetics) and a final formulation (extraction procedures used), our findings are most relevant to our investigated oil-based formulations and may not be applicable to the safety of other marketed formulations consisting of a different profile of cannabinoids and other cannabis constituents (e.g., terpenes) and delivered in a different matrix (e.g., not in an oil formulation). Our study was also a preliminary safety study and, as such, a small number of animals were used, which is a limitation. Additional larger studies that investigate the safety of longer-term cannabinoid dosing in dogs are needed.

Overall, our study provides novel data that separates the relative safety and tolerability of dose escalation of oil formulations predominant in plant-derived CBD, THC, or CBD and THC in combination (1.5:1) in dogs. Of the three cannabinoid oil formulations tested, dose escalation of the CBD-predominant oil formulation was the most tolerated by dogs up to a maximum dose of 640.5 mg CBD (~62 mg CBD/kg) and only mild AEs were experienced. Novel data on the *in vivo* metabolism of CBD vs. THC when delivered at higher dose levels were generated, which showed that CBD is absorbed more slowly than THC.

Research on the potential health benefits of CBD in dogs is beginning to emerge. Existing studies show its potential as a single therapy for pain reduction in osteoarthritic dogs (17) or as an adjunct therapy for the reduction in seizure frequency in dogs with idiopathic epilepsy (21). Our findings provide support for continuing research on CBD's safety profile and potential therapeutic uses in dogs so that it may be considered a treatment option in veterinary medicine.

## Data Availability Statement

The datasets generated for this study are not publicly available to allow for commercialization of research findings. Reasonable requests to access the datasets should be directed to Phil Shaer (phil.shaer@canopygrowth.com).

## Ethics Statement

All animal care and experimental procedures were conducted under protocols approved by the facility's Institutional Animal Care and Use Committee (IACUC) and in accordance with the Principles of the Animals for Research Act (53) and guidelines of the Canadian Council on Animal Care (CCAC).

## Author Contributions

DV and JK were responsible for conception of the study, data analysis, and provided intellectual input on the manuscript. LP was responsible for data analysis and interpretation and writing of the manuscript.

### Conflict of Interest

DV, JK, and LP are employed by Canopy Animal Health, which is a division of Canopy Growth Corporation. Staff at VivoCore Inc., and not the authors, were responsible for study conduct and data collection.
